# Identifying Alzheimer’s Disease Progression Subphenotypes Via a Graph-based Framework Using Electronic Health Records

**DOI:** 10.1007/s41666-026-00230-2

**Published:** 2026-02-11

**Authors:** Yu Huang, Jie Xu, Zhengkang Fan, Yu Hu, Xing He, Aokun Chen, Yuxi Liu, Rui Yin, Jingchuan Guo, Steven T. DeKosky, Michael Jaffee, Manqi Zhou, Chang Su, Fei Wang, Yi Guo, Jiang Bian

**Affiliations:** 1 Department of Biostatistics and Health Data Science, Indiana University School of Medicine, Indianapolis, IN 46202 USA; 2 Center for Biomedical Informatics, Regenstrief Institute, Indianapolis, IN 46202 USA; 3 Department of Health Outcomes & Biomedical Informatics, University of Florida, Gainesville, FL 32610 USA; 4 Pharmaceutical Outcomes & Policy, University of Florida, Gainesville, FL 32603 USA; 5 Department of Neurology and McKnight Brain Institute, University of Florida, Gainesville, FL 32610 USA; 6 Department of Neurology, Norman Fixel Institute for Neurological Diseases, University of Florida, Gainesville, FL 32608 USA; 7 Department of Computational Biology, Cornell University, Ithaca, NY 14853 USA; 8 Department of Population Health Sciences, Weill Cornell Medicine, New York, NY 10065 USA; 9 Institute of Artificial Intelligence for Digital Health, Weill Cornell Medicine, New York, NY 10065 USA; 10 Indiana University Health, Indianapolis, IN 46290 USA

**Keywords:** Alzheimer’s disease, Disease progression subphenotyping, Real-world data, Graph neural network, Electronic health records

## Abstract

Understanding the heterogeneity of neurodegeneration in Alzheimer’s disease (AD) and identifying distinct progression pathways is critical for improving diagnosis, treatment, prognosis, and prevention. Motivated by this need, this study aimed to identify disease progression subphenotypes among patients with mild cognitive impairment (MCI) and AD using electronic health records (EHRs). We developed a novel approach that combines a graph neural network (GNN)–based framework with time series clustering to characterize progression subphenotypes from MCI to AD. We applied the proposed framework to a real-world cohort of 2,525 patients (61.66% female; mean age 76 years), of whom 64.83% were Non-Hispanic White, 16.48% Non-Hispanic Black, 2.53% were of other races, and 10.85% were Hispanic. Our model identified four distinct progression subphenotypes, each exhibiting characteristic clinical patterns, with average MCI-to-AD progression times ranging from 805 to 1,236 days. These findings indicate that AD does not follow a uniform progression trajectory but instead manifests heterogeneous pathways, and the proposed framework provides an explainable, data-driven approach for delineating AD progression subphenotypes, offering actionable insights for healthcare informatics research and the clinical management of patients at risk for AD.

**Supplementary Information:**

The online version contains supplementary material available at 10.1007/s41666-026-00230-2.

## Introduction

Alzheimer’s disease (AD) is the most common form of dementia, characterized by a gradual and irreversible decline in cognitive function. This heterogeneous aging-related neurodegenerative disorder affects a large number (approximately 1 in 9 people aged 65) of older adults globally [1]. As of 2023, an estimated 6.7 million Americans are living with AD, and this number is projected to increase to 13.85 million by 2060 [1, 2]. The growing population of individuals affected by AD will undoubtedly impose a substantial burden on patients, their families, the healthcare system, and society at large, presenting a critical problem that requires careful consideration and attention.

The AD continuum, which describes the progression of AD, starts with brain changes that often go unnoticed and advances in the majority of cases to memory difficulties, ultimately leading to significant cognitive and functional impairments. This progression is commonly hypothesized to encompass three broad phases: preclinical AD, clinically significant mild cognitive impairment (MCI) due to AD, and Alzheimer’s dementia [3–5]. The duration of each phase of AD varies among individuals, influenced by factors such as demographics, genetics, environmental factors, and lifestyle, leading to heterogeneous clinical outcomes [6]. The rate of cognitive decline varies widely among AD patients; some experience rapid deterioration, while others show slower rates of decline over time [7]. Therefore, understanding the variety of progression patterns within the AD continuum and the ability to identify early diagnostic indicators are of significant clinical importance.

Recent studies have focused on incorporating diverse data sources to study the development of AD, including clinical variables [8], neuroimaging [9, 10], neuropsychological data [11, 12], and neuropathological data [13], aiming to identify clinical predictors and biomarkers [14–16] that can be used to track disease changes [17]. Disease subphenotyping that segments patients diagnosed with the same disease into multiple sub-entities (subphenotypes), with unique clinical manifestations, phenotypic progression trajectories, and/or clinical outcomes, has been attracting increasing attention in biomedicine to study complex diseases like AD [18]. Existing studies have often focused on a restricted range of features, such as neuroimaging [19], neuropsychological data [12], and neuropathological data [13], to identify subphenotypes of AD patients. However, some of these data sources (e.g., neuroimaging biomarkers) require specialized and costly equipment that are not widely available. This limitation restricts their use in routine clinical care in less well-equipped settings and constrains the sample size available for subphenotyping analyses.

The proliferation of accessible real-world data (RWD), especially electronic health records (EHRs) and administrative claims data, coupled with advancements in artificial intelligence (AI) and machine learning (ML) techniques, has opened new avenues for investigating the heterogeneity of AD progression in a data-driven manner [20]. Longitudinal EHRs collected during routine care provide fine-grained encounter information, enabling tracking of changes in the health status of AD patients. These records are valuable for identifying AD subphenotypes, yet few studies have focused on utilizing them for this purpose [21–24]. Most existing approaches do not consider relevant clinical outcomes, such as disease severity, leading to limited utility for clinicians and patients. Additionally, these methods have struggled to capture progression characteristics in the AD continuum, and they have been limited in modeling the similarity of progression trends and patterns among patients. Therefore, it is necessary to model the underlying relationships among patient encounters with the health systems and introduce outcome-oriented learning approaches [25] to identify subgroups (i.e., subphenotypes) with homogeneous progression and clinical characteristics that are strongly associated with future clinical outcomes.

We developed a novel computational framework combining a graph neural network (GNN) with time series clustering for disease progression subphenotyping. This framework leverages a directed patient graph (DPG) to model patient longitudinal EHRs, connecting visits with similar clinical characteristics and enabling GNNs to effectively learn patient progression patterns. We incorporated an outcome-driven strategy to guide the GNN, ensuring that the extracted embeddings are clinically relevant to the progression from MCI to AD. Additionally, this framework utilizes time series clustering to analyze sequential embeddings learned from GNNs, identifying MCI to AD progression subphenotypes (i.e., pathways). Through a series of experiments conducted on a large-scale EHR cohort from the OneFlorida + Clinical Research Consortium, we demonstrated the presence of different progression subphenotypes leading to AD. In addition, we evaluated whether information available up to the time of MCI diagnosis can predict a patient’s membership in these progression subphenotypes, thereby assessing their potential utility for early risk stratification. This study fills important gaps in understanding the heterogeneity of AD progression, which can potentially enhance earlier diagnosis and intervention for AD.

## Methods

### Data Source and Study Population

**Fig. 1:**
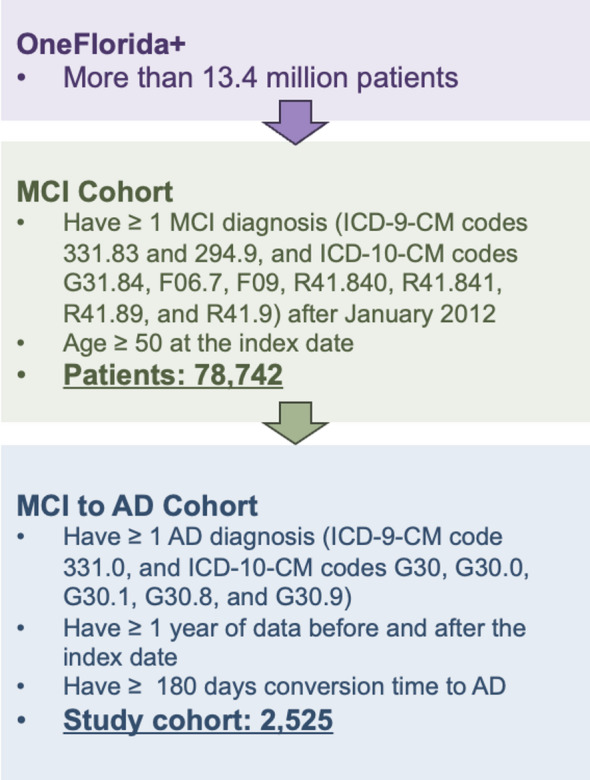
Overview of the study cohort extracted from the OneFlorida + network

In this study, we used RWD from OneFlorida+, a clinical research network contributing to the national Patient-Centered Clinical Research Network (PCORnet), which collaborates with a group of 14 health organizations. OneFlorida + contains robust, longitudinal, patient-level EHRs of 16.8 million patients from Florida, 2.1 million from Georgia, and 1.1 million from Alabama, and is linked at the patient level with various other data sources, including selected Medicaid and Medicare claims, vital statistics, and tumor registries. OneFlorida + is a HIPAA-limited data set (i.e., dates and 9-digit zip codes are available) following the PCORnet Common Data Model (CDM) and contains detailed patient and clinical variables, including demographics, vital signs, conditions, diagnoses, procedures, prescribing, dispensing, and lab results, among others, covering data from 2012 to 2023. Figure 1 shows an overview of the study cohort construction process. The study cohort includes patients who: (1) had one of the following MCI diagnosis codes (International Classification of Diseases, Ninth Revision, Clinical Modification [ICD-9-CM] codes 331.83 or 294.9, and International Classification of Diseases, Tenth Revision, Clinical Modification [ICD-10-CM] codes G31.84, F06.7, F09, R41.840, R41.841, R41.89, or R41.9), and (2) were 50 years or older at the time of their first MCI diagnosis. We further identified patients with AD within the MCI cohort who had the following ICD-9-CM code 331.0 or ICD-10-CM codes G30, G30.0, G30.1, G30.8, G30.9. Those who had AD diagnosis codes before their MCI diagnosis were excluded from the study. To ensure a sufficient duration of data for training the proposed framework, we imposed two additional inclusion criteria: (1) patients were required to have at least one year of data before and after the index date, and (2) patients were required to have a conversion time to AD of more than six months.

### Disease Progression Subphenotyping Framework

**Fig. 2:**
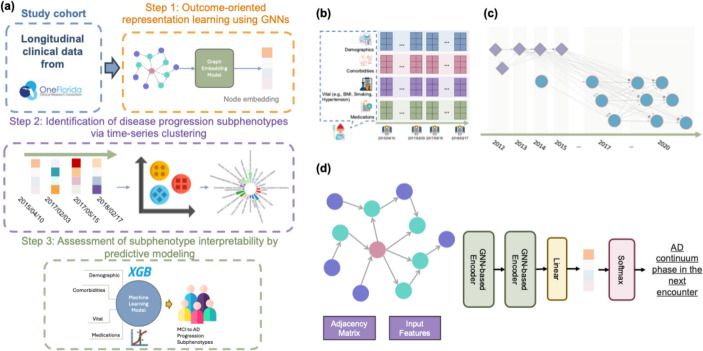
Overview of the disease progression subphenotyping framework. **a** Study pipeline. XGB denotes the XGBoost model. **b** Example of an EHR sequence for a patient, where each visit record includes demographics, comorbidities, vital signs, and medications. **c** Illustration of the proposed disease progression graph (DPG), in which each node corresponds to an enhanced encounter representation $\:{\widehat{x}}_{t}^{n}$; node shape and color distinguish patients, and dotted lines indicate edges linking nodes across different patients. **d** Graph neural network (GNN)-based framework for learning embeddings of individual data points

Figure 2-a illustrates the proposed framework, which consists of three components: (1) Outcome-oriented representation learning using GNNs, (2) Identification of disease progression subphenotypes via time-series clustering, and (3) Assessment of subphenotype interpretability by predictive modeling.

#### Step 1: Outcome-oriented Representation Learning Using GNNs

The longitudinal EHRs of the 𝑛-th patient can be represented as $\:\left\{{x}_{t}^{n}\right\}$, where $\:t\:\in\:\{\mathrm{1,2},\dots\:,\mathrm{T}\}$, and $\:{x}_{t}^{n}$contains multi-source information (e.g., demographics, diagnoses, and treatments) documented at each visit (encounter). For each encounter, we first discretized age using uniform-sized bins and utilized one-hot encoding to encode variables such as age, gender, and race-ethnicity. We then mapped the diagnosis codes (i.e., ICD-9-CM and ICD-10-CM codes from the raw EHRs) to Phecodes [26], which are designed to support phenome-wide association studies (PheWAS) in EHRs, and transformed the drug codes (e.g., National Drug Codes [NDC] and RxNorm) to the third level of the Anatomical Therapeutic Chemical (ATC) Classification System. We discretized the Body Mass Index (BMI) into four categories: underweight (≤ 18.5), normal weight (18.5–24.9), overweight (25–29.9), and obesity (≥ 30) [27], the blood pressure readings to five classes (normal, elevated, hypertension stage 1, hypertension stage 2, and hypertensive crisis) [28], and smoking status to current, former, non-smoker, and others. Finally, we formed a binary vector by concatenating the encoded age, gender, and race from the encounters with diagnoses and medications in a three-month period up to time point $\:t$ for each patient, resulting in an enhanced encounter representation $\:{\widehat{x}}_{t}^{n}$ representing the patient’s current clinical status at that time. Each $\:{\widehat{x}}_{t}^{n}$ was then assigned a label for presenting the patient’s health status of the next visit period: Pre-MCI (the period prior to the first documented MCI diagnosis), MCI, or AD (identified by ICD codes mentioned in the section *Data source and study population*). Specifically, we included patient demographics, medications, labs, and diagnosis codes within each three-month window as input features and predicted the patient’s status in the following three-month window. This design avoids label leakage because the diagnoses correspond to the patient’s current disease state, not the future label being predicted. We term this approach outcome-oriented representation learning; the diagnoses reflect the evolving clinical profile of the patient, which may either remain stable or progress to the next stage. Importantly, our primary objective is not the short-term prediction itself, but rather to leverage this setup as a self-supervised mechanism for learning robust longitudinal patient representations. By conditioning on diagnosis codes at each stage, the model better captures evolving temporal dynamics and provides a more faithful embedding of disease trajectories (e.g., transitions from Pre-MCI to MCI to AD). These representations are intended to support downstream analyses of disease progression, rather than to serve solely as point predictions of the immediate next time window.

Then, we modeled the processed longitudinal EHRs (Fig. 2-b) into a novel disease progression graph (DPG). This graph structure effectively captures individual patient progression patterns and preserves the inter-patient progression correlations, as depicted in Fig. 2-c. Formally, we defined the DPG as a directed graph, $\:DPG=\left(\mathcal{V},\mathcal{\:}\mathcal{E},\mathcal{A}\right)$, where each node $\:v\in\:\mathcal{V}$ represents an enhanced encounter representation $\:{\widehat{x}}_{t}^{n}$, incorporating patient characteristics (e.g., demographics, diagnoses, vital information, and treatments). To build the DPG, we identified the top-k most similar neighbors for each node using Jaccard index [29], which is well-suited since the encounter features are presented as binary vectors. Subsequently, we inserted a directed edge $\:e\in\:\mathcal{E}$ between each pair of similar nodes. In addition, we checked and inserted edges between nodes (i.e., enhanced encounter representations) from the same patient to ensure that the progression of the disease can be linked through a path. These connections could span across patients and within patients, with edge direction reflecting the chronological order of encounters (i.e., edges only went from earlier visits to later ones). The adjacency matrix $\:\mathcal{A}$ captures the weights of the edges (i.e., the elapsed time between two encounters).

To generate outcome-oriented embeddings for each node, we developed an encoder based on GNNs as shown in Fig. 2-d. This encoder utilizes the graph structures to propagate and aggregate node features in an iterative manner. The encoder contains two GNN layers and a fully connected layer to transform the learned embeddings to a specific label. The GNN layers [30] can be described as:

$$\:{h}_{v}=\:\varphi\:\left({F}_{v},\underset{u\in\:{N}_{v}}\oplus\psi\:({F}_{v},{F}_{u},{a}_{v,u})\right)$$

where $\:{F}_{v}$ refers to the original features of node $\:v$, $\:{h}_{v}$ refers to the learned embeddings for a node $\:v$, $\:\varphi\:$ and $\:\psi\:$ are learnable functions, $\oplus$ is a nonparametric operation (e.g., aggregation and concatenation), $\:{N}_{v}$ means the neighbors of $\:v$, and $\:{a}_{v,u}$ is the weight of the edge between nodes $\:v$ and $\:u$. We utilized MagNet [31], a kind of graph convolutional network (GCN) designed for directed graphs, as the core of the GNN layer. MagNet extends traditional GCNs by incorporating directional information via a complex Hermitian matrix. With its superior ability to encode structural information from directed graphs and outperforming traditional GCNs on various benchmarks, MagNet is an excellent candidate for modeling the intricate information of disease progression contained within DPGs. Additionally, we implemented other three variants of GNNs, including GCN [32], graph attention network (GAT) [33], and graph sample and aggregate network (GraphSAGE) [34] to compare their performance in embedding learning.

The embeddings for each node are generated by GNN layers, capturing the underlying disease progression information relevant to patient encounters. These outcome-oriented embeddings are then fed into the fully connected predictor, which forecasts the outcomes of the next encounter along the AD continuum: preclinical, MCI, and AD. The fully connected layer serves as a prediction head, and prediction loss trains the entire model end-to-end, updating the GNN parameters in an outcome-oriented approach. The fully connected layer can be formulated as:

$$\:y=\theta\:(W{h}_{v}+b)$$

where $\:\theta\:$ is the activation function and $\:W$ and $\:b$ are the learnable parameters. During the training stage, we employed gradient descent to optimize the GNNs by minimizing the difference between the actual and predicted labels.

#### Step 2: Identification of Disease Progression Subphenotypes Via time-series Clustering

After obtaining the learned embeddings of each node (i.e., enhanced encounter representation), we combined these embeddings into a sequence (forming a multivariate time series). Each patient had an embedding sequence arranged in chronological order based on the original longitudinal EHRs, described as $\:{H}^{n}=\{{h}_{1}^{n},\dots\:,\:{h}_{T}^{n}\}$, where $\:n$ and T are the patient and time index, respectively. We then applied the time-series K-means clustering method with dynamic time warping (DTW) [35] to identify similar latent characteristics among embedding sequences and determine progression subphenotypes. We chose time series K-means due to its simplicity as an unsupervised algorithm, known for its rapid convergence, even on large datasets. Time series K-means clusters similar time series data by grouping them based on their similarity. This algorithm repeatedly reassigns time series to clusters and updates the centroids to minimize the within-cluster errors, as defined:

$$\:J=\sum\:_{i=1}^{K}\sum\:_{j=1}^{{C}_{i}}DTW({H}_{\left(j\right)}^{\left(i\right)},{\mu\:}_{i})$$

where $\:K$ is the number of clusters, $\:{C}_{i}$ refers to the number of patients in cluster $\:i$, $\:{H}_{\left(j\right)}^{\left(i\right)}$ is the embedding sequence of the $\:j$-th patient in cluster $\:i$, and $\:{\mu\:}_{i}$ denotes the centroid of cluster $\:i$.

Since patient embedding sequences vary in length due to different visit histories, we adopted DTW to measure similarity between two embedding sequences of unequal length. For example, consider two patient embedding sequences (patient n and m): $\:{H}^{n}=\{{h}_{1}^{n},\dots\:,\:{h}_{T}^{n}\}$ with corresponding indices $\:S=\left\{{S}_{1},\dots\:,{S}_{N}\right\}$, and $\:{H}^{m}=\{{h}_{1}^{m},\dots\:,\:{h}_{T{\prime\:}}^{m}\}$ with corresponding indices $\:{S}^{{\prime\:}}=\left\{{{S}^{{\prime\:}}}_{1},\dots\:,{{S}^{{\prime\:}}}_{M}\right\}$. The goal of DTW is to find an optimal warping path with the minimum cumulative distance between $\:S$ and $\:{S}^{{\prime\:}}$. A warping path is defined as a sequence $\:P=\{{p}_{1},\dots\:{p}_{L}\}$, where $\:{p}_{l}=\left({n}_{l},{m}_{l}\right)$ represents an alignment between $\:{S}_{nl}$ and $\:{{S}^{{\prime\:}}}_{ml}$ with $\:\mathrm{n}\mathrm{l}\in\:\{1,...,N\}$ and $\:\mathrm{m}\mathrm{l}\in\:\{1,...,M\}$. The length of the path, denoted by $\:L$, corresponds to the total number of aligned index pairs $\:\left({n}_{l},{m}_{l}\right)$.

Formally, the cumulative cost of a path $\:P$ is defined as:

$$\:{D}_{P}(S,S{\prime\:})=\sum\:_{l=1}^{L\in\:P}d({S}_{nl},{S{\prime\:}}_{ml})$$

where $\:d\left(\right)$ is the distance function (e.g., Euclidian distance) between two data points in different sequences. The DTW distance is then given by:

$$\:{DTW}\left(S,{S}^{{\prime\:}}\right):={D}_{{P}^{*}}\left(S,{S{\prime\:}}\right)=\underset{P\in\:D(S,{S{\prime\:}})}{\mathrm{min}}\left\{{D}_{P}(S,S{\prime\:})\right\}$$

where $\:D(S,S{\prime\:})$ is the set of all possible warping paths, and $\:{P}^{*}$ is the optimial path.

#### Step 3: Assessment of Subphenotype Interpretability by Predictive Modeling

To ensure that the subphenotypes were clinically useful, we built prediction models to assess their predictability. We first generated the subphenotypes based on the proposed GNN framework for each patient. Then, we defined the prediction index date as each patient’s first MCI diagnosis date. For the prediction models, we constructed a cross-sectional feature vector for each patient by aggregating information (e.g., demographics, comorbidities, and medications) from their first visit in the database up to the index date to predict which longitudinal progression pattern (subphenotype) a patient is likely to follow. No information after the MCI diagnosis date was used as predictor input. We implemented various commonly used ML models for this purpose, including linear models (e.g., logistic regression [36], lasso regression [37], ridge regression [38], and ElasticNet [39]) and XGBoost [40–45]. We also incorporated two imbalanced data preprocessing methods, random over sampling and random under sampling. Finally, we utilized SHapley Additive exPlanations (SHAP) [46] – a widely used XAI technique – to identify important features contributing to the models’ ability to classify the subphenotypes.

### Modeling Procedures and Benchmarks

To learn the patient representations using GNN, we followed ML best practices, stratified the data by patients, and split it into training, validation, and testing sets according to a 70%:10%:20% ratio. We additionally conducted 10-fold cross-validation experiments, which consistently demonstrated stable performance across folds. We selected the Area under the Receiver Operating Characteristic Curve (AUROC) as our primary metric and included sensitivity, specificity, and precision as additional metrics for the GNNs and the subphenotype prediction model. Furthermore, we conducted a five-fold cross-validation Bayesian search on the training set to optimize the parameters of the subphenotype prediction models.

To determine the optimal $\:K$ in the clustering analysis, we combined quantitative and qualitative evaluations. Specifically, we applied the time series K-means algorithm with varying cluster numbers (K = 2 to 10) to generate clustering outcomes under different K settings. Then, we used silhouette score (SS) [47] and Davies-Bouldin Index (DBI) [48], with DTW as the similarity metric, to measure the quality of clusters. Clustering results were considered acceptable if SS was above 0.25 and DBI was below 1 [49]. To validate cluster robustness and stability, we conducted extensive bootstrapping experiments (100 iterations) with repeated random splits and clustering initializations. In each iteration, time series k-means was trained on 90% of the data and tested on the full dataset, and the resulting cluster assignments were compared against the full-sample solution using Jaccard similarity, SS, and DBI. After we obtained the candidate results (e.g., K from 2 to 4 in MagNet showing fair and acceptable quantitative performance), we examined the characteristics of each cluster under different settings and manually assessed cluster quality. The qualitative assessment focused on (1) variations in the transition time from MCI to AD, (2) differences in survival time post-AD diagnosis, and (3) comorbidities, medications, and demographics. Two reviewers (Y.H. and J.X.) independently evaluated cluster quality and ranked each candidate as low, medium, or high. Then, a consensus discussion was led by a third reviewer (J.B.) to consolidate opinions and determine the optimal K [50].

### Subphenotype Analysis

We evaluated clinical outcomes across AD progression subphenotypes using time-to-event survival analysis with the Kaplan–Meier estimator (lifelines library) [51]. For mortality, time was measured as the number of days from first AD diagnosis to death recorded in the EHRs, with patients without a recorded death date treated as right-censored at their last known follow-up. For disease progression, transition from MCI to AD was analyzed using time from first MCI diagnosis to first AD diagnosis, where individuals who did not convert during follow-up were right-censored at their last recorded visit. Differences in survival probability and AD-conversion rate across subphenotypes were visualized using stratified Kaplan–Meier curves with 95% confidence intervals.

## Results

### Descriptive Statistics of the Study Cohort

Our final analysis included 2,525 eligible MCI and AD patients in the cohort. eTable 1 highlights the characteristics of the study cohort. The mean age of the patients was 76 (std = 8.88), with 61.66% being women. In the cohort, 64.83% were Non-Hispanic White (NHW), 16.48% were Non-Hispanic Black (NHB), and 2.53% were of other races. Additionally, there were 274 Hispanic patients, accounting for 10.85% of the total patient population. The average duration from their first MCI diagnosis to transition to AD was 891 days.

### GNN Performance for Learning Disease Progression Representation

 eTables 2 to 5 present the performance analysis of four GNNs under different settings, including the number of neighbors (*n* = 25,50,100,200) in DPG, choice of loss functions (e.g., cross-entropy or Focal loss [52]) and the embedding size (e.g., 32 or 64) for learning outcome-oriented disease progression representations. The GCN model shows the lowest performance, with all metrics around or below 0.9. In contrast, MagNet, GAT, and GraphSAGE demonstrated exemplary performance, with consistently high precision, recall, and specificity rates, all exceeding 0.95. Notably, increasing the dimensionality of the embeddings correlated with a slight improvement in model prediction performance. Among the two loss functions, Focal loss achieved better performance metrics. To sum up, our model utilized a total of 2,067 input features after one-hot encoding: 13 demographic variables, 1,852 comorbidities, 187 medication indicators, and 15 vital signs. We acknowledge that this represents a high-dimensional feature space relative to the available patient cohort. To address this concern, we have now conducted 10-fold cross-validation experiments (eTable 6) in addition to our original train/validation/test split. The results remain consistent with our initial findings, showing that the predictive performance of the MagNet model (accuracy, recall, and specificity > 0.95) is stable across folds. This strengthens our confidence that the observed performance is not an artifact of a single partition but rather reflects the model’s ability to generalize.

### Identifying AD Progression Subphenotypes

**Fig. 3:**
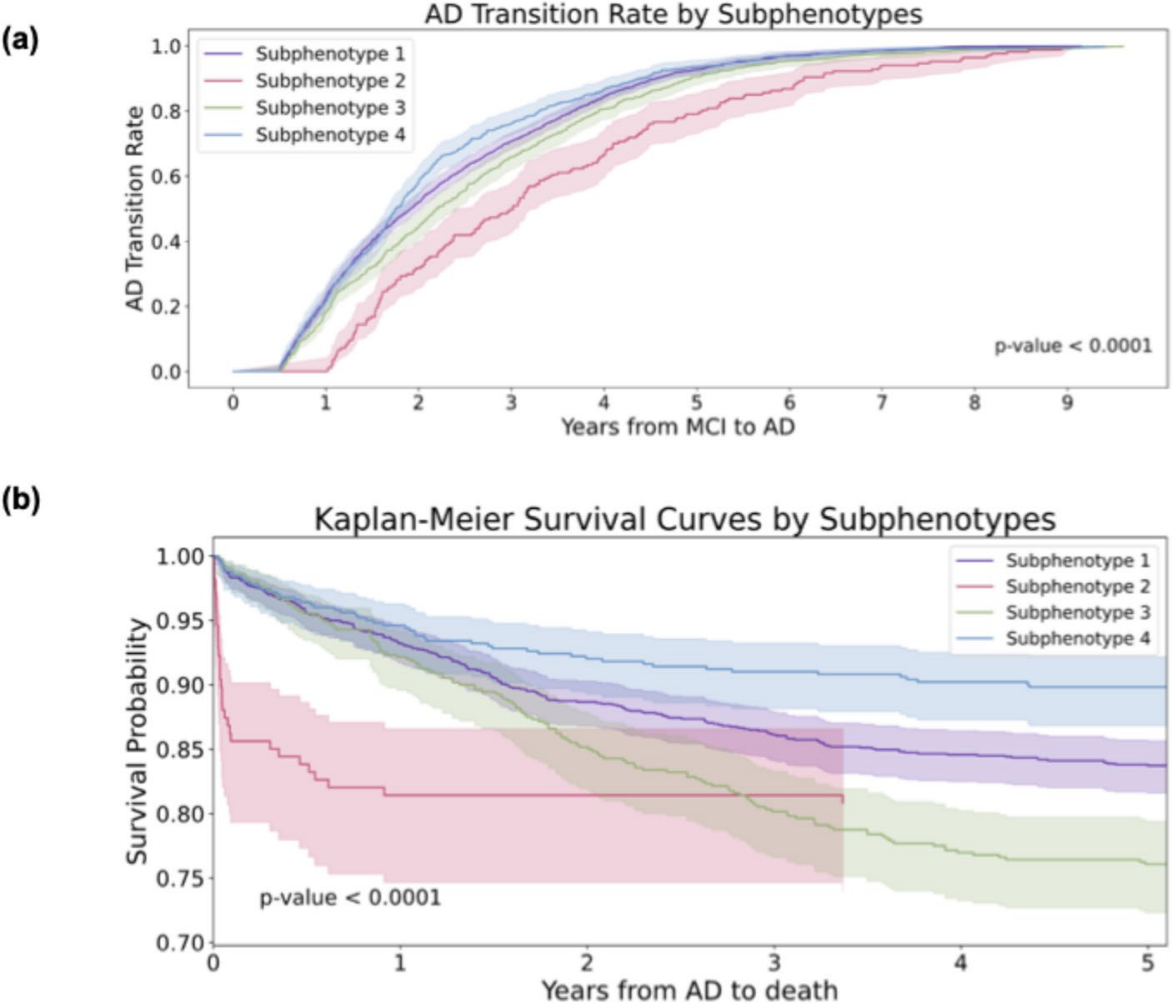
Visualization of Mild Cognitive Impairment (MCI) to Alzheimer’ Disease (AD) progression subphenotype. **a** MCI to AD transition rates by subphenotypes. **b** Kaplan-Meier survival curves by subphenotypes

**Fig. 4:**
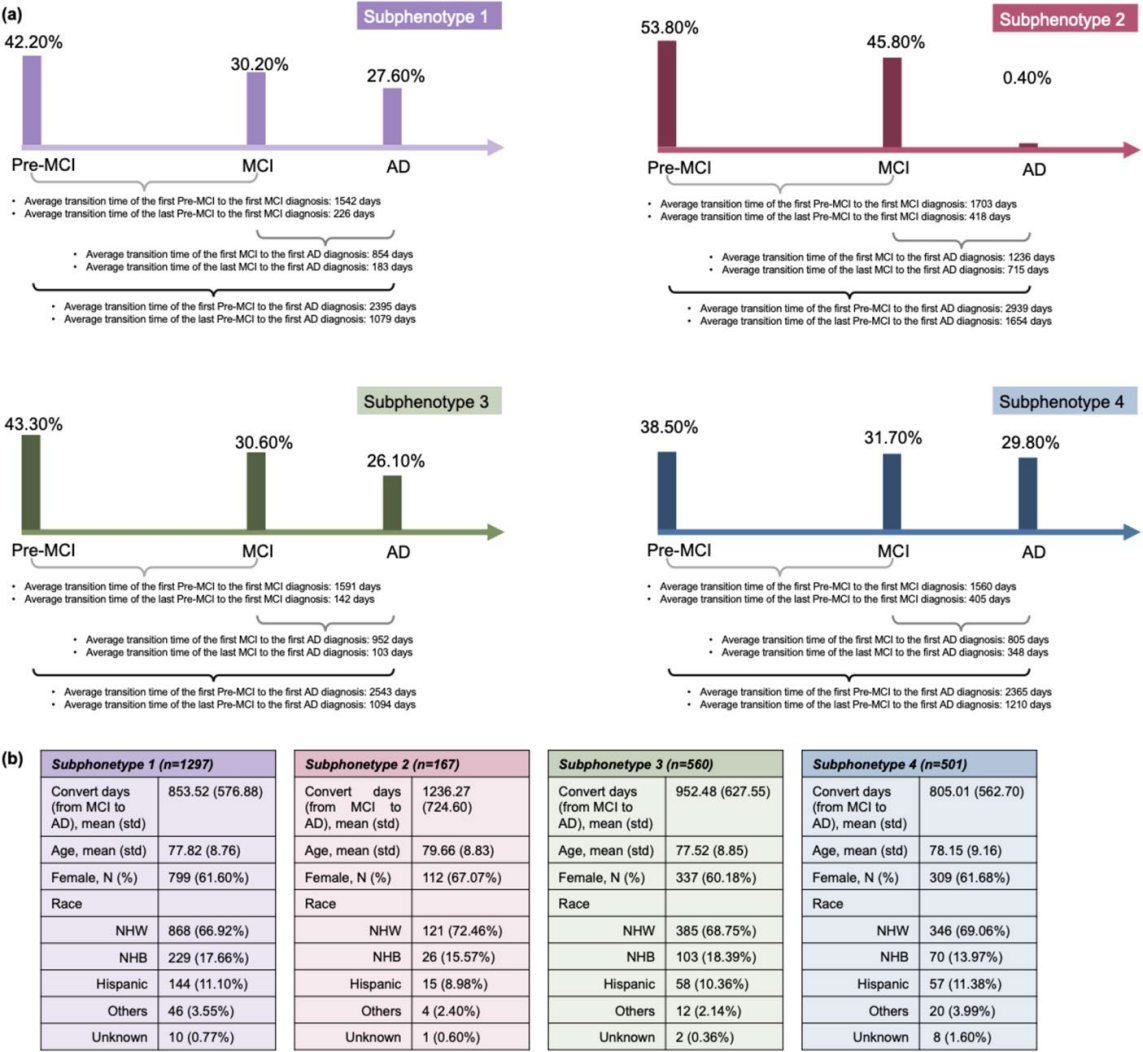
Characteristics of the AD progression subphenotypes. **a** Average transition times across the stages of AD in the four subphenotypes. Percentages above the bars indicate the proportion of a single encounter in each category (Pre-MCI, MCI, and AD). **b** Demographics of each subphenotype

Through the K-selection procedure (identifying the number of clusters) that combined quantitative and qualitative analyses (details of the model selection are provided in Supplement Sect. 1; eTables 2–6 present the GNN model performance comparisons, eFigure 1 reports the SS and DBI metrics across K variations, eTable 7 reports the clustering stability, and eFigs. 2, 3, 4, 5, 6, 7, 8, 9, 10, 11, 12 and 13 describe the detailed characteristics of the candidate subphenotypes), we determined that the optimal number of clusters was four (K = 4). Figure 3-a shows the transition rates and days from MCI to AD across subphenotypes. Figure 3-b visualizes the Kaplan-Meier curve, showing the 5-year survival rates for each subphenotype after AD diagnosis. There is a notable difference (*p* < 0.001) among the various subphenotypes. Figure 4-a shows detailed AD progression information among phenotypes. Patients in subphenotype 1 (S1) and S4 are characterized by faster progression, with average times from the initial record to the first AD diagnosis being 2,395 days and 2,365 days, respectively. Conversely, individuals in S2 and S3 exhibit a more stable and slower progression to AD, with average transition times from the first EHR record to the first AD diagnosis being 2,939 days and 2,543 days, respectively. Figure 4-b illustrates the demographic statistics for each subphenotype; S1 includes a higher number of patients (*n* = 1,297) than the others, while the patients in these four subphenotypes share comparable demographic distributions. When considering the MCI to AD transition rate, S1 (mean: 854 days; std: 577 days) and S4 (mean: 805 days; std: 563 days) progress more rapidly than S2 (mean: 1,236 days; std: 725 days) and S3 (mean: 952 days; std: 628 days), however, with large variations within each subphenotype.

**Fig. 5:**
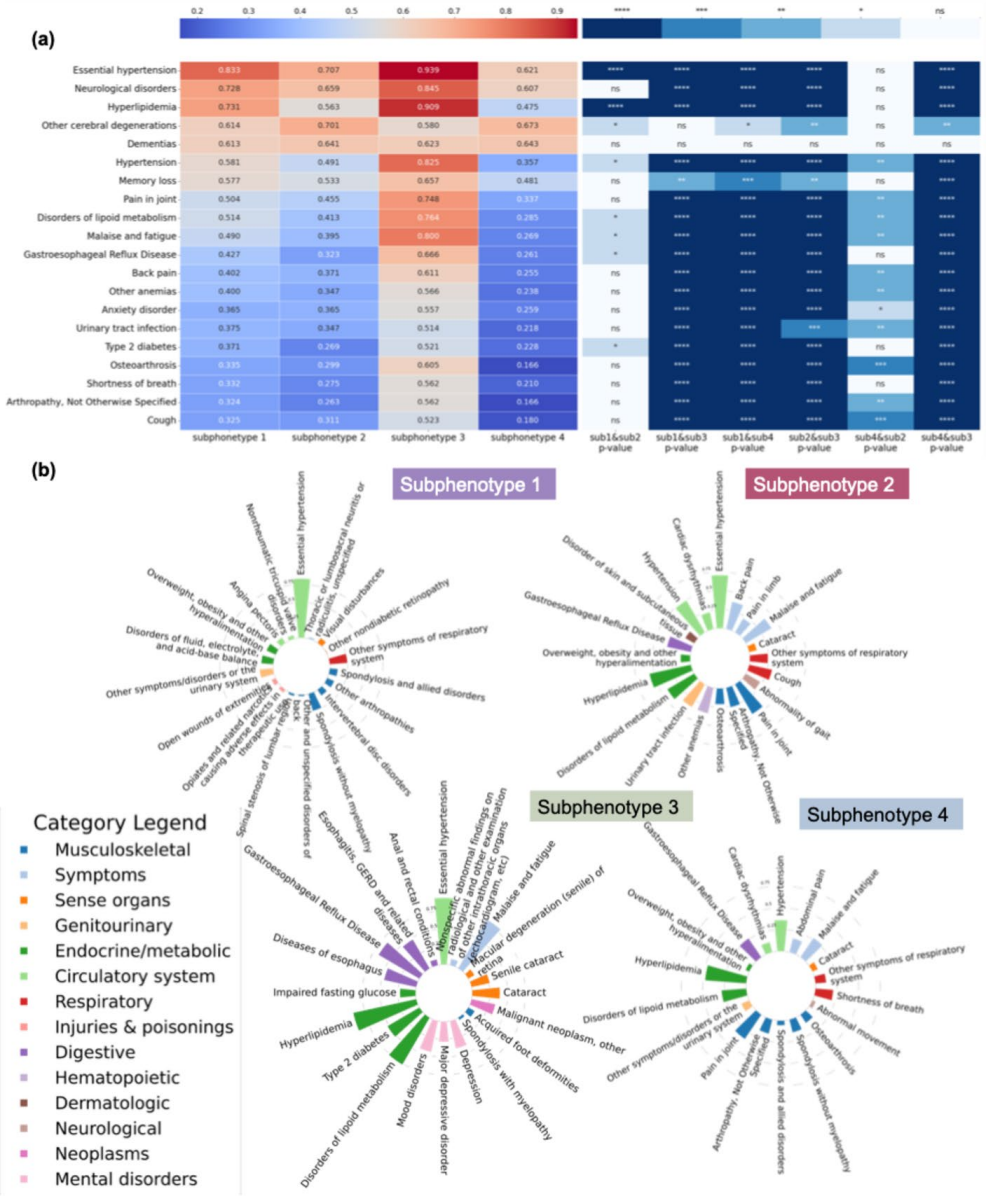
Clinical characteristics of the AD progression subphenotypes. **a** Heatmap of the top 20 features with the highest prevalence in the cohort across the four subphenotypes, along with p-value comparisons between each pair of subphenotypes. To simplify the visualization, p-values are represented by asterisks: *p* < 0.01 (*), *p* < 0.001 (**), *p* < 0.0001 (***), and *p* < 0.00001 (****); ns indicates non-significance. **b** Most correlated comorbidities and their prevalence for each subphenotype

Figure 5 plots the unique clinical characteristics of each cluster. To gain insights into the features differentiating these subphenotypes, we first selected the top 20 features with a high percentage in the study cohort and then examined their prevalence within each subphenotype (left panel of Fig. 5-a). The analysis revealed that essential hypertension was the most prevalent disease among all the subphenotypes. Additionally, S4 included patients with lower prevalence, while S3 comprised patients with higher prevalence. We performed statistical analysis using Chi-square tests to examine the significant differences between pairs of subphenotypes. The corresponding features vs. subphenotype p-values (e.g., hypertension/subtypes 1 to 4) are presented in the right panel of Fig. 5-a. We computed pairwise Pearson correlations among the four subphenotypes using prevalence-based features (eTable 8). Notably, significant differences emerged between most paired subphenotypes, though S1 and S2 showed relatively high similarity. Additionally, in Fig. 5-b, we highlight the top features (filtered by p-value < 0.05) for each subphenotype, and the bars indicate the prevalence of specific factors within each subphenotype. Diseases of the circulatory system were common among all subphenotypes; overlapping features were observed, particularly conditions related to the musculoskeletal and endocrine/metabolic systems.

Across Figs. 3, 4 and 5, S1 and S4, the faster progression subphenotypes, were linked to common comorbidities associated with AD, including cardiovascular, gastrointestinal, and musculoskeletal diseases. S2 and S3 showed relatively slow AD progression but had lower survival rates within five years after an AD diagnosis, with a higher prevalence and variety of associated comorbidities.

### Predictability and Interpretability of the Identified Subphenotypes

**Fig. 6:**
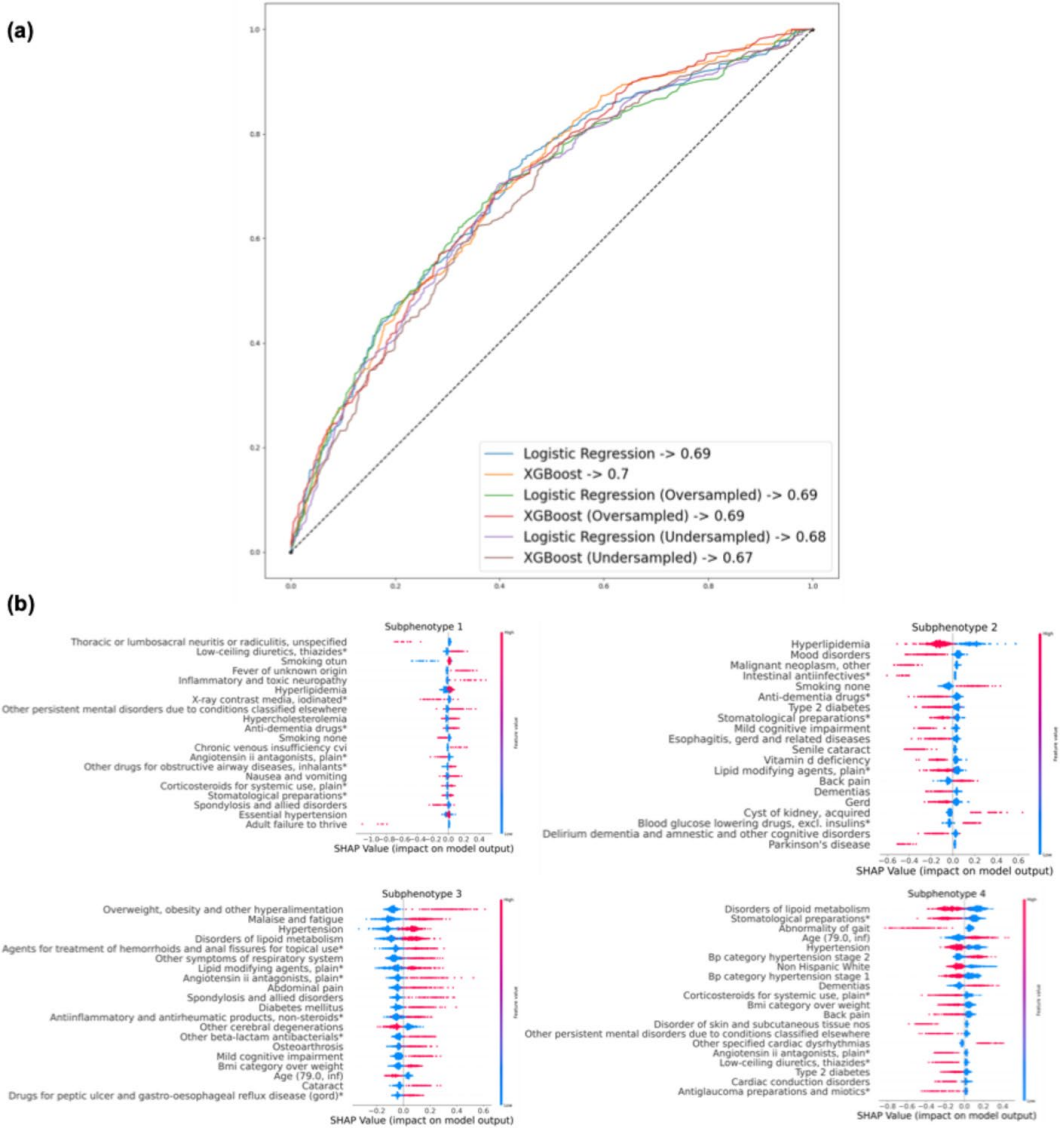
Comparative assessment of the predictability and interpretability of XGBoost and linear models in predicting AD subphenotypes. **a** Model performance for predicting progression subphenotypes based on encounter information. **b** Analysis of feature importance in XGBoost using SHAP values

Figure 6-a shows the performance of prediction models that classify patients into one of the four subphenotypes using information available before the MCI diagnosis date, aiming to evaluate whether information available up to the time of MCI diagnosis can predict a patient’s membership in these progression subphenotypes, thereby assessing their potential utility for early risk stratification. We evaluated multiple algorithms (e.g., linear models vs. XGBoost) and resampling strategies (e.g., oversampling vs. undersampling) to address data imbalance. For these models, predictors (e.g., demographics, comorbidities, and medications) were extracted from their first visit up to the index date and then aggregated into a cross-sectional representation. These predictors are based on the same underlying clinical concepts as the variables used in the GNN model; however, the GNN operates on their full longitudinal, visit-level structure to learn progression patterns, whereas the prediction models use only the pre-MCI aggregated information to predict the patient’s subsequent progression subphenotype.

Detailed results are provided in Supplement Sect. 2 (see eTable 9). Our results showed that all models delivered fair performance, with Area Under the Receiver Operating Characteristic (AUROC) ranging from 0.67 to 0.7, where XGBoost showed comparable predictive performance to logistic regression. Regarding resampling methods, non-resampling, oversampling, and undersampling all yielded similar results. Figure 6-b presents the SHAP values of the XGBoost model, which predicts the likelihood of patients being classified into different subphenotypes. Across the four subphenotypes, the key factors influencing classification include the patient’s age, existing neurological conditions (e.g., memory loss), and current dementia stage (e.g., Pre-MCI, MCI, or dementia due to AD). Specifically focusing on S4, considered the most rapid progression subphenotype, the model underlined the fact that being older than 79, experiencing hypertension stage 2 [53, 54], and being on dementia medications are key predictors of being in this subphenotype, consistent with findings in the existing literature [55].

## Discussion

We developed a novel outcome-oriented GNN framework that naturally (1) models sequences of events in longitudinal EHRs as directed graphs, and (2) captures similar patient encounters through directed edges. This framework enables GNNs to generate representations across patients with similar characteristics while considering the changes in individual patients’ health conditions. Utilizing time-series K-means clustering on the representations learned from GNNs, our approach can effectively model nuanced similarities in disease progression patterns. We used large collections of EHRs from the OneFlorida + network and identified 2,525 patients with MCI over an observation period of up to 10 years (eTable 1). Our results demonstrated that our proposed framework holds promise in identifying predictable AD progression subphenotypes, providing valuable and explainable insights into the development of the disease.

Several studies (Table 1) have focused on disease progression subphenotyping. However, most of this work either does not incorporate relevant clinical outcomes (e.g., the disease continuum) or is limited in modeling the similarities in progression trends and patterns among patients. As a result, these approaches struggle to effectively capture disease progression characteristics, reducing their utility for both clinicians and patients. For example, Xu et al. proposed an LSTM-based framework to define progression states and identified two distinct progression patterns within MCI cohorts, without considering progression pathways in similar patients [22]. Song et al. introduced the DisEase PrOgression Trajectory (DEPOT) approach to model cancer-related chronic kidney disease (CKD) progression trajectories from electronic medical records [56]. Nagamine et al. employed a cluster-based approach to understand the real-world manifestation and progression of heart failure by constructing disease states from clinical notes [57]. Additionally, Chowdhury et al. encoded longitudinal patient EHRs into a graph structure and applied a graph transformer for drug response prediction. The learned embeddings from the graph transformer were then used to stratify patients into subgroups [58]. In contrast to these existing methods, our framework leverages a DPG to model patients’ longitudinal EHRs as a directed graph, linking visits with similar clinical characteristics, which serves as a foundation for GNNs to learn comprehensive patient progression patterns. Furthermore, our framework adopts an outcome-oriented strategy to guide the representation learning process, ensuring that the extracted features are both clinically meaningful and predictive of the progression from MCI to AD. By integrating these key components, our method enhances the interpretability of disease progression, offering deeper insights into patient trajectories and supporting more informed clinical decision-making.

**Table 1:** Relevant study comparison

	Disease Focus	Key method		Outcomes
		Representation Learning	Clustering	
Xu et al. [22]	Alzheimer’s disease (AD) progression	Long short-term memory-based deep learning	Hierarchical Agglomerative Clustering	Identify AD progression states and summarize progression patterns
Song et al. [56]	Chronic kidney disease (CKD) progression	GraphSAGE	DDRTree	Maps diverse CKD progression paths and cancer risks
Nagamine et al. [57]	Heart Failure	NLP feature extraction	K-Means	Identify heart failure disease states
Chowdhury et al. [58]	Heart Failure drug response	Graph Neural Network + Transformer	K-Means	Identifies HF subtypes with differential drug responses
Ours	Mild cognitive impairment (MCI) to AD progression	Outcome-oriented Graph neural networks	Time series K-Means	Identify MCI to AD progression subphenotypes

Using the GNN-based framework and time-series clustering, we identified four subphenotypes of patients with distinct progression patterns from MCI to AD. These patterns suggest that AD does not follow uniform transitions of disease states but rather exhibits heterogeneous progression pathways, aligning with the existing research [59–61]. For instance, Geifman et al. [59] identified three clinical phenotypes of AD from clinical trials, each following a distinct trajectory: slow decline, severely impaired yet slow decline, or rapid decline. However, this study did not explore the clinical characteristics of these subtypes in detail, such as the correlation between comorbidities and each subtype. In contrast, in 2023, Xu et al.[22] defined two main distinct progressions within the MCI cohorts—one leading to AD and one that did not. The pathway leading to AD was notable for significant differences in symptoms such as memory loss, various dementias, and articular cartilage disorders, which are typical in AD cases. Garg et al. [62] characterized the progression from MCI to AD into four categories with different health conditions using AD cohort study data from the Mayo Clinic Study of Aging (MCSA). Compared to these studies, our framework identified four progression subphenotypes from MCI to AD from routine care records, providing detailed clinical characteristics of each, including comorbidities across various systems like nervous, musculoskeletal, cardiovascular, alimentary tract and metabolic, genitourinary, and sensory systems. We also outlined the distinct rates of progression for each subphenotype. A better understanding of these distinct disease progression patterns could help the exploration of more personalized and potentially effective treatment strategies, potentially slowing or preventing their progression to AD. Our approach can not only help identify (1) patients at a higher risk of AD progression, years before they reach the clinical stage, where early-stage treatments [63, 64], such as anti-amyloid drugs aducanumab and lecanemab (approved by the FDA for targeting AD’s underlying biology) might be more effective, but also (2) their potential disease progression pathways, so that providers and patients can better plan potential treatment strategies (e.g., the management of comorbidities and symptoms).

The subphenotypes we identified, showcasing variations in traits linked to disorders—primarily in musculoskeletal, circulatory, endocrine/metabolic, digestive, and sensory systems, as well as in their progression rates, provide important insights. Hypertension [53, 54], often considered a risk factor for cognitive decline and AD, is tied to all progressing subphenotypes. Disorders related to lipoprotein metabolism [65] and symptoms such as malaise and fatigue were associated with three subphenotypes. Studies [66, 67] suggested that targeting APOE [68, 69], a key factor in lipid metabolism, could lead to developing treatments that may help AD patients. Managing symptoms, for example, through the therapeutic use of nicotinamide adenine dinucleotide (NAD) [70], holds the potential to benefit patients with AD as well as those experiencing chronic fatigue syndrome. The rapidly progressing subphenotypes S1 and S4 were mainly associated with musculoskeletal and cardiovascular conditions, including cardiac arrhythmias [71–73]. Managing cardiac arrhythmias could potentially slow cognitive decline in AD by reducing the risk of strokes and other cardiovascular complications[73]. Furthermore, due to a lower prevalence of comorbidities compared to S2 and S3, these subphenotypes also exhibited lower five-year survival rates. For the stable progression subphenotype (S2), in addition to common comorbidities, we found associations with conditions such as urinary tract infections (UTIs) [74, 75], hematopoietic disorders, and neurological diseases. UTIs, in particular, were linked to cognitive impairments in individuals with MCI or AD, often manifesting as delirium and exacerbating dementia symptoms. However, treating UTIs may lead to some cognitive improvements. Our findings indicate that S3, a subgroup with a high prevalence of mental health conditions (e.g., mood disorders and depression), digestive disorders (e.g., esophageal diseases [76]), and neoplasms, exhibited a slower AD progression rate. However, while S2 and S3 demonstrated more stable AD progression patterns, their high prevalence of multiple comorbidities contributed to lower five-year survival rates compared to other subphenotypes. Understanding these diverse subphenotypes provides valuable insights into different patient subgroups with distinct characteristics. This knowledge can be leveraged for post-trial analysis, optimizing patient recruitment, and assessing drug effectiveness.

### Strengths and Limitations

Our study has several significant clinical implications. First, the proposed framework enables a more precise AD diagnosis by identifying unique progression pathways, each characterized by distinct clinical factors such as comorbidities and medication use. Second, recognizing various progression subphenotypes is very valuable for understanding the disease’s progression in AD patients, offering potential prognoses of AD, which is vital for informing future care and support planning. Finally, since each subphenotype may respond differently to treatments, discerning and testing these differences allows more targeted and effective therapeutic options if these findings are confirmed in future trials, thus aiding in developing personalized treatment strategies.

Our study has several limitations and opportunities for future exploration. First, the analysis conducted in our study was based on a cohort of MCI and AD patients mainly in Florida, Georgia, and Alabama; this limited geographical scope might affect the generalizability of our findings. However, our real-world AD patients from OneFlorida + were highly diverse, including both rural and urban populations, and reflected the demographic changes (a high prevalence of racial-ethnic) minorities, occurring across the US. The subphenotypes identified in this study were derived using an unsupervised, data-driven approach in a single cohort. As with other clustering-based phenotyping studies, there is no external gold standard against which to judge the “correctness” of the clusters, and the resulting subgroups are inherently dependent on the study population, variable selection, and modeling assumptions. We therefore view the proposed subphenotypes as hypothesis-generating rather than definitive clinical entities. Although we assessed their robustness and clinical plausibility by demonstrating consistent differences in MCI to AD progression that were not used to define the clusters, we did not validate the model in an independent dataset. Future research should apply the proposed method across different cohorts and institutions to validate the findings and the generalizability of the method. Nevertheless, future research should aim to enhance our proposed framework’s generalizability utilizing data from different geographic regions. Second, while dominantly inherited Alzheimer’s disease (DIAD) could theoretically affect analysis outcomes, its impact is minimal. In our study using RWD, without genetic or biomarker data we cannot completely exclude rare DIAD cases, but DIAD accounts for < 1% of AD [77], differs mainly in age at onset, family history, and co-pathologies, and shares similar clinical features with common AD [78]. Given our study cohort of diagnoses after age 50, the likelihood of including DIAD—typically presenting earlier—is very low, making bias unlikely. Third, examining causes of death across subphenotypes could provide valuable insights. Yet, because our PCORnet CDM EHR data lack this information, we could not assess mortality causes. Fourth, for identifying AD progression subphenotypes from patient embedding sequences, we selected Time series K-Means with DTW, which offered a practical compromise between scalability and simplicity for our exploratory objectives. Models such as HDBSCAN, EM Alpha-Stable Mixture Models, and Spectral Clustering could be tailored for time series analysis and warrant further exploration in future research. Fifth, while the AUROC indicates an acceptable level of performance, other metrics suggest only fair predictive capability. Predicting progression subphenotypes remains a challenging task, and traditional models such as linear models and XGBoost struggle to achieve high accuracy. Additionally, the current subphenotyping prediction model is data-driven. Considering a knowledge-driven modeling approach may improve subphenotype prediction methods.

## Conclusion

We proposed a novel outcome-oriented GNN framework for identifying AD progression subphenotypes using EHRs. The four subphenotypes suggest that AD exhibits heterogeneous progression pathways rather than following uniform transitions of disease states. These subphenotypes provide valuable and explainable insights for the development of AD.

## Supplementary Information

Below is the link to the electronic supplementary material.

Supplementary Material 1

## Data Availability

The data presented in this study are available on request from the corresponding author. The data are not publicly available due to privacy restrictions.

## Code Availability

The codes presented in this study are available on request from the corresponding author.
