# Supplementary material for: Identifying Alzheimer’s Disease Progression Subphenotypes Via a Graph-based Framework Using Electronic Health Records
Source: J Healthc Inform Res. 2026 Feb 11;10(2):317–39. doi: 10.1007/s41666-026-00230-2 (PMC13128099; doi:10.1007/s41666-026-00230-2)
Supplement: Supplementary file 1 — Supplementary Material 1 [file 41666_2026_230_MOESM1_ESM.docx]

**Supplementary Materials**

This supplementary file contains the additional figures and tables of the experimental results for readers. The content is outlined as follows:

- **Supplement Section 1:** Details of the model selection
- **Supplement Section 2:** Details of the predictability and interpretability of the identified subphenotypes
- **eTable 1.** Characteristics of the study cohort
- **eTable 2.** Predictive performance of various GNNs (number of neighbors = 25) on the AD continuum phase for patient follow-up encounters
- **eTable 3.** Predictive performance of various GNNs (number of neighbors = 50) on the AD continuum phase for patient follow-up encounters
- **eTable 4.** Predictive performance of various GNNs (number of neighbors = 100) on the AD continuum phase for patient follow-up encounters
- **eTable 5.** Predictive performance of various GNNs (number of neighbors = 200) on the AD continuum phase for patient follow-up encounters
- **eTable 6.** 10-fold Cross-validation to evaluate predictive performance of various GNNs on the AD continuum phase for patient follow-up encounters
- **eTable 7.** Bootstrapped Clustering Results (100 Iterations)
- **eTable 8.** Pairwise Pearson correlations among the four subphenotypes
- **eTable 9.** Model performance (XGBoost) for predicting subphenotypes based on encounter information
- **eFigure 1.** Silhouette score and Davies-Bouldin Index for time series k-means clustering (K = 2 to 10) utilizing embeddings from GAT, GraphSAGE, and MagNet.
- **eFigure 2.** Characteristics of AD progression subphenotypes identified by MagNet with time series K-means (K = 3). (a) MCI to AD transition rates for each subphenotype. (b) Kaplan-Meier survival curves stratified by subphenotype. (c) Demographic characteristics of each subphenotype.
- **eFigure 3.** Clinical characteristics of AD progression subphenotypes identified by MagNet with time series K-means (K = 3). (a) Heatmap of the top 20 features with the highest prevalence in the cohort across the subphenotypes, along with p-value comparisons between each pair of subphenotypes. (b) Most correlated comorbidities and their prevalence for each subphenotype.
- **eFigure 4.** Characteristics of AD progression subphenotypes identified by GraphSAGE with time series K-means (K = 3). (a) MCI to AD transition rates for each subphenotype. (b) Kaplan-Meier survival curves stratified by subphenotype. (c) Demographic characteristics of each subphenotype.
- **eFigure 5.** Clinical characteristics of AD progression subphenotypes identified by GraphSAGE with time series K-means (K = 3). (a) Heatmap of the top 20 features with the highest prevalence in the cohort across the subphenotypes, along with p-value comparisons between each pair of subphenotypes. (b) Most correlated comorbidities and their prevalence for each subphenotype.
- **eFigure 6.** Characteristics of AD progression subphenotypes identified by GraphSAGE with time series K-means (K = 4). (a) MCI to AD transition rates for each subphenotype. (b) Kaplan-Meier survival curves stratified by subphenotype. (c) Demographic characteristics of each subphenotype.
- **eFigure 7.** Clinical characteristics of AD progression subphenotypes identified by GraphSAGE with time series K-means (K = 4). (a) Heatmap of the top 20 features with the highest prevalence in the cohort across the subphenotypes, along with p-value comparisons between each pair of subphenotypes. (b) Most correlated comorbidities and their prevalence for each subphenotype.
- **eFigure 8.** Characteristics of AD progression subphenotypes identified by GraphSAGE with time series K-means (K = 5). (a) MCI to AD transition rates for each subphenotype. (b) Kaplan-Meier survival curves stratified by subphenotype. (c) Demographic characteristics of each subphenotype.
- **eFigure 9.** Clinical characteristics of AD progression subphenotypes identified by GraphSAGE with time series K-means (K = 5). (a) Heatmap of the top 20 features with the highest prevalence in the cohort across the subphenotypes, along with p-value comparisons between each pair of subphenotypes. (b) Most correlated comorbidities and their prevalence for each subphenotype.
- **eFigure 10.** Characteristics of AD progression subphenotypes identified by GraphSAGE with time series K-means (K = 6). (a) MCI to AD transition rates for each subphenotype. (b) Kaplan-Meier survival curves stratified by subphenotype. (c) Demographic characteristics of each subphenotype.
- **eFigure 11.** Clinical characteristics of AD progression subphenotypes identified by GraphSAGE with time series K-means (K = 6). (a) Heatmap of the top 20 features with the highest prevalence in the cohort across the subphenotypes, along with p-value comparisons between each pair of subphenotypes. (b) Most correlated comorbidities and their prevalence for each subphenotype.
- **eFigure 12.** Characteristics of AD progression subphenotypes identified by GraphSAGE with time series K-means (K = 7). (a) MCI to AD transition rates for each subphenotype. (b) Kaplan-Meier survival curves stratified by subphenotype. (c) Demographic characteristics of each subphenotype.
- **eFigure 13.** Clinical characteristics of AD progression subphenotypes identified by GraphSAGE with time series K-means (K = 7). (a) Heatmap of the top 20 features with the highest prevalence in the cohort across the subphenotypes, along with p-value comparisons between each pair of subphenotypes. (b) Most correlated comorbidities and their prevalence for each subphenotype.

**Supplement Section 1: Details of the model selection**

From **eTables 2 to 5**, we observed that MagNet, GAT, and GraphSAGE demonstrated strong performance. To further validate model stability, we conducted 10-fold cross-validation, and the consistent results in **eTable 6** supported the findings from **eTables 2–5**. These consistent results show that the predictive performance of the MagNet, GAT, and GraphSAGE models is stable. Across the four neighborhood sizes (25, 50, 100, 200), model performance does not show a consistent monotonic trend with respect to the number of neighbors. GAT and GraphSAGE remain highly stable and near-optimal across all neighbor settings, while MagNet generally benefits from larger neighborhoods (peaking around 100–200 neighbors) and GCN tends to degrade as the neighborhood size increases. Since their performances were comparable, we selected the best-performing parameters for each GNN model:

- MagNet: #neighbors = 200, cross-entropy loss, embedding dimension = 64
- GAT: #neighbors = 50, cross-entropy loss, embedding dimension = 64
- GraphSAGE: #neighbors = 50, focal loss, embedding dimension = 32

These models were then used to generate embeddings for the next phase of clustering experiments.

**eFigure 1** presents the Silhouette Score (SS) and Davies-Bouldin Index (DBI) for time series k-means clustering (K = 2 to 10) using embeddings from GAT, GraphSAGE, and MagNet. We found that only GraphSAGE (K = 3 to 7) and MagNet (K = 3 to 4) met our quantitative criteria (SS > 0.25 and DBI < 1). To further assess clustering stability, we performed bootstrapping. **eTable 7** reports the results from 100 bootstrap iterations for each clustering outcome. In each iteration, time series k-means was trained on 90% of the data with random sampling, tested on 100%, and the Jaccard overlap was compared with the full-sample run. SS and DBI values are presented as averages across the 100 iterations. These results indicate that clustering stability is acceptable, with Jaccard scores ranging from 0.6 to 0.75,^1^ suggesting that the clusters capture meaningful patterns in the data. The bootstrap results for SS and DBI were also consistent with those in **eFigure 1**, further confirming clustering robustness. To further analyze the subphenotypes, we plotted their detailed characteristics for GraphSAGE (K = 3 to 7) and MagNet (K = 3 to 4) in **eFigures 2 to 13**. Considering key factors such as: (1) Variations in the transition time from MCI to AD, (2) Differences in survival time post-AD diagnosis, and (3) Comorbidities, medications, and demographics, we ultimately selected MagNet with time series k-means (K = 4) as the final model to generate MCI to AD progression subphenotypes. This section complements the experiments by providing detailed results for the 'Identifying AD progression subphenotypes' section in the main text.

**Supplement Section 2: Details of the predictability and interpretability of the identified subphenotypes**

**eTable 9** presents the detailed performance metrics (precision, recall, and F1-score) of the best-performing prediction model, XGBoost, designed to classify one of the four subphenotypes based on data extracted by the MCI diagnosis. While the AUROC indicates an acceptable level of performance, other metrics suggest only fair predictive capability. Predicting progression subphenotypes remains a challenging task, and traditional models such as linear models and XGBoost struggle to achieve high accuracy. This section complements the experiments by providing detailed results for the 'Predictability and Interpretability of the Identified Subphenotypes' section in the main text.

**eTable 1.** Characteristics of the study cohort

|  | Study cohort (N = 2,525, patients diagnosed with MCI who developed to AD) |
| --- | --- |
| Observation period, mean days (std) | 3,166 (831) |
| Characteristics |  |
| Conversion days from MCI to AD, mean (std) | 891 (605) |
| Age, mean (std) | 75.5 (8.88) |
| Female, N (%) | 1,557 (61.66%) |
| Race-ethnicity, N (%) |  |
| Non-Hispanic White | 1,637 (64.83%) |
| Non-Hispanic Black | 416 (16.48%) |
| Hispanic | 274 (10.85%) |
| Others | 64 (2.53 %) |
| Unknown | 134 (5.30%) |

**eTable 2.** Predictive performance of various GNNs (number of neighbors = 25) on the AD continuum phase for patient follow-up encounters

|  | **Model** | **Accuracy** | **F1** | **Precision** | **Recall** | **AUROC** | **Specificity** |
| --- | --- | --- | --- | --- | --- | --- | --- |
| **Cross-entropy**  **Output dim = 32** | MagNet | 0.949 | 0.944 | 0.946 | 0.942 | 0.958 | 0.975 |
|  | GCN | 0.812 | 0.802 | 0.805 | 0.800 | 0.853 | 0.905 |
|  | GAT | 0.969 | 0.967 | 0.970 | 0.965 | 0.974 | 0.984 |
|  | GraphSAGE | 0.960 | 0.958 | 0.962 | 0.956 | 0.968 | 0.980 |
| **Cross-entropy**  **Output dim = 64** | MagNet | 0.966 | 0.964 | 0.967 | 0.962 | 0.972 | 0.983 |
|  | GCN | 0.811 | 0.801 | 0.804 | 0.799 | 0.852 | 0.905 |
|  | GAT | 0.969 | 0.967 | 0.970 | 0.965 | 0.974 | 0.984 |
|  | ***GraphSAGE*** | 0.960 | 0.957 | 0.958 | 0.955 | 0.968 | 0.980 |
| **Focal Loss**  **Output dim = 32** | MagNet | 0.927 | 0.923 | 0.929 | 0.921 | 0.943 | 0.964 |
|  | GCN | 0.804 | 0.785 | 0.791 | 0.787 | 0.844 | 0.901 |
|  | GAT | 0.968 | 0.966 | 0.968 | 0.963 | 0.973 | 0.983 |
|  | GraphSAGE | 0.965 | 0.963 | 0.965 | 0.962 | 0.972 | 0.982 |
| **Focal Loss**  **Output dim = 64** | MagNet | 0.910 | 0.898 | 0.916 | 0.895 | 0.925 | 0.955 |
|  | GCN | 0.793 | 0.781 | 0.780 | 0.782 | 0.840 | 0.897 |
|  | GAT | 0.968 | 0.966 | 0.968 | 0.964 | 0.974 | 0.984 |
|  | GraphSAGE | 0.968 | 0.965 | 0.967 | 0.964 | 0.974 | 0.983 |

**eTable 3.** Predictive performance of various GNNs (number of neighbors = 50) on the AD continuum phase for patient follow-up encounters

|  | **Model** | **Accuracy** | **F1** | **Precision** | **Recall** | **AUROC** | **Specificity** |
| --- | --- | --- | --- | --- | --- | --- | --- |
| **Cross-entropy**  **Output dim = 32** | MagNet | 0.962 | 0.958 | 0.960 | 0.957 | 0.969 | 0.981 |
|  | GCN | 0.749 | 0.724 | 0.727 | 0.729 | 0.801 | 0.873 |
|  | GAT | 0.967 | 0.965 | 0.968 | 0.962 | 0.973 | 0.983 |
|  | GraphSAGE | 0.958 | 0.955 | 0.956 | 0.954 | 0.966 | 0.979 |
| **Cross-entropy**  **Output dim = 64** | MagNet | 0.966 | 0.963 | 0.966 | 0.961 | 0.972 | 0.982 |
|  | GCN | 0.761 | 0.739 | 0.743 | 0.743 | 0.811 | 0.879 |
|  | **GAT** | **0.972** | **0.971** | **0.973** | **0.968** | **0.977** | **0.986** |
|  | *GraphSAGE* | 0.944 | 0.942 | 0.941 | 0.942 | 0.957 | 0.972 |
| **Focal Loss**  **Output dim = 32** | MagNet | 0.946 | 0.940 | 0.944 | 0.938 | 0.955 | 0.973 |
|  | GCN | 0.748 | 0.731 | 0.732 | 0.732 | 0.803 | 0.875 |
|  | GAT | 0.956 | 0.953 | 0.955 | 0.952 | 0.965 | 0.978 |
|  | **GraphSAGE** | **0.970** | **0.968** | **0.971** | **0.966** | **0.975** | **0.985** |
| **Focal Loss**  **Output dim = 64** | MagNet | 0.961 | 0.957 | 0.962 | 0.955 | 0.968 | 0.980 |
|  | GCN | 0.745 | 0.723 | 0.723 | 0.728 | 0.800 | 0.872 |
|  | GAT | 0.967 | 0.965 | 0.968 | 0.963 | 0.973 | 0.983 |
|  | GraphSAGE | 0.969 | 0.967 | 0.970 | 0.966 | 0.975 | 0.984 |

**eTable 4.** Predictive performance of various GNNs (number of neighbors = 100) on the AD continuum phase for patient follow-up encounters

|  | **Model** | **Accuracy** | **F1** | **Precision** | **Recall** | **AUROC** | **Specificity** |
| --- | --- | --- | --- | --- | --- | --- | --- |
| **Cross-entropy**  **Output dim = 32** | MagNet | 0.963 | 0.961 | 0.963 | 0.960 | 0.971 | 0.981 |
|  | GCN | 0.717 | 0.689 | 0.691 | 0.696 | 0.776 | 0.857 |
|  | GAT | 0.960 | 0.957 | 0.961 | 0.955 | 0.967 | 0.979 |
|  | GraphSAGE | 0.953 | 0.950 | 0.951 | 0.950 | 0.963 | 0.977 |
| **Cross-entropy**  **Output dim = 64** | MagNet | 0.968 | 0.965 | 0.968 | 0.963 | 0.973 | 0.983 |
|  | GCN | 0.718 | 0.693 | 0.693 | 0.702 | 0.781 | 0.860 |
|  | GAT | 0.967 | 0.965 | 0.968 | 0.962 | 0.973 | 0.983 |
|  | GraphSAG***E*** | 0.955 | 0.952 | 0.953 | 0.951 | 0.964 | 0.977 |
| **Focal Loss**  **Output dim = 32** | MagNet | 0.960 | 0.957 | 0.960 | 0.955 | 0.967 | 0.980 |
|  | GCN | 0.714 | 0.681 | 0.685 | 0.690 | 0.772 | 0.855 |
|  | GAT | 0.956 | 0.954 | 0.957 | 0.951 | 0.964 | 0.977 |
|  | GraphSAGE | 0.967 | 0.965 | 0.967 | 0.963 | 0.973 | 0.983 |
| **Focal Loss**  **Output dim = 64** | MagNet | 0.965 | 0.962 | 0.966 | 0.959 | 0.971 | 0.982 |
|  | GCN | 0.723 | 0.693 | 0.696 | 0.701 | 0.780 | 0.860 |
|  | GAT | 0.970 | 0.968 | 0.971 | 0.966 | 0.975 | 0.985 |
|  | GraphSAGE | 0.970 | 0.968 | 0.970 | 0.966 | 0.975 | 0.984 |

**eTable 5.** Predictive performance of various GNNs (number of neighbors = 200) on the AD continuum phase for patient follow-up encounters

|  | **Model** | **Accuracy** | **F1** | **Precision** | **Recall** | **AUROC** | **Specificity** |
| --- | --- | --- | --- | --- | --- | --- | --- |
| **Cross-entropy**  **Output dim = 32** | MagNet | 0.969 | 0.967 | 0.969 | 0.965 | 0.975 | 0.984 |
|  | GCN | 0.687 | 0.656 | 0.660 | 0.663 | 0.752 | 0.841 |
|  | GAT | 0.955 | 0.950 | 0.957 | 0.948 | 0.962 | 0.977 |
|  | GraphSAGE | 0.961 | 0.958 | 0.960 | 0.956 | 0.968 | 0.980 |
| **Cross-entropy**  **Output dim = 64** | **MagNet** | **0.971** | **0.969** | **0.972** | **0.967** | **0.976** | **0.985** |
|  | GCN | 0.693 | 0.669 | 0.668 | 0.674 | 0.760 | 0.846 |
|  | GAT | 0.971 | 0.970 | 0.972 | 0.967 | 0.976 | 0.985 |
|  | *GraphSAGE* | 0.945 | 0.940 | 0.941 | 0.941 | 0.957 | 0.973 |
| **Focal Loss**  **Output dim = 32** | MagNet | 0.969 | 0.967 | 0.970 | 0.965 | 0.975 | 0.984 |
|  | GCN | 0.686 | 0.657 | 0.659 | 0.662 | 0.751 | 0.841 |
|  | GAT | 0.957 | 0.954 | 0.958 | 0.951 | 0.965 | 0.978 |
|  | GraphSAGE | 0.963 | 0.961 | 0.962 | 0.960 | 0.970 | 0.981 |
| **Focal Loss**  **Output dim = 64** | MagNet | 0.963 | 0.960 | 0.964 | 0.957 | 0.969 | 0.981 |
|  | GCN | 0.692 | 0.663 | 0.664 | 0.670 | 0.757 | 0.845 |
|  | GAT | 0.952 | 0.949 | 0.953 | 0.945 | 0.960 | 0.975 |
|  | GraphSAGE | 0.960 | 0.958 | 0.960 | 0.956 | 0.968 | 0.980 |

**eTable 6.** 10-fold Cross-validation to evaluate predictive performance of various GNNs on the AD continuum phase for patient follow-up encounters

| **#Nbr** | **Loss** | **#Dim** | **Model** | **Accuracy** | **F1** | **Precision** | **Recall** | **AUROC** | **Specificity** |
| --- | --- | --- | --- | --- | --- | --- | --- | --- | --- |
| 25 | Cross-entropy Loss | 32 | MagNet | 0.928 (± 0.039) | 0.919 (± 0.044) | 0.931 (± 0.034) | 0.917 (± 0.043) | 0.941 (± 0.031) | 0.964 (± 0.018) |
|  |  |  | GCN | 0.818 (± 0.017) | 0.805 (± 0.02) | 0.809 (± 0.022) | 0.805 (± 0.017) | 0.857 (± 0.012) | 0.909 (± 0.008) |
|  |  |  | GAT | 0.971 (± 0.004) | 0.969 (± 0.005) | 0.972 (± 0.005) | 0.967 (± 0.004) | 0.976 (± 0.003) | 0.985 (± 0.002) |
|  |  |  | GraphSAGE | 0.962 (± 0.007) | 0.959 (± 0.007) | 0.961 (± 0.008) | 0.958 (± 0.007) | 0.969 (± 0.005) | 0.981 (± 0.003) |
|  |  | 64 | MagNet | 0.909 (± 0.05) | 0.897 (± 0.057) | 0.912 (± 0.054) | 0.895 (± 0.055) | 0.925 (± 0.04) | 0.955 (± 0.026) |
|  |  |  | GCN | 0.819 (± 0.013) | 0.806 (± 0.015) | 0.812 (± 0.019) | 0.804 (± 0.013) | 0.857 (± 0.01) | 0.909 (± 0.006) |
|  |  |  | GAT | 0.97 (± 0.005) | 0.967 (± 0.006) | 0.97 (± 0.007) | 0.965 (± 0.006) | 0.975 (± 0.004) | 0.985 (± 0.002) |
|  |  |  | GraphSAGE | 0.961 (± 0.007) | 0.958 (± 0.008) | 0.959 (± 0.009) | 0.956 (± 0.007) | 0.968 (± 0.005) | 0.98 (± 0.003) |
|  | Focal Loss | 32 | MagNet | 0.909 (± 0.036) | 0.895 (± 0.045) | 0.919 (± 0.025) | 0.893 (± 0.044) | 0.924 (± 0.031) | 0.955 (± 0.018) |
|  |  |  | GCN | 0.812 (± 0.015) | 0.797 (± 0.015) | 0.805 (± 0.015) | 0.795 (± 0.016) | 0.85 (± 0.012) | 0.906 (± 0.007) |
|  |  |  | GAT | 0.967 (± 0.008) | 0.965 (± 0.007) | 0.968 (± 0.008) | 0.963 (± 0.007) | 0.973 (± 0.006) | 0.983 (± 0.004) |
|  |  |  | GraphSAGE | 0.966 (± 0.01) | 0.963 (± 0.01) | 0.966 (± 0.01) | 0.961 (± 0.01) | 0.972 (± 0.008) | 0.983 (± 0.005) |
|  |  | 64 | MagNet | 0.842 (± 0.091) | 0.821 (± 0.112) | 0.862 (± 0.081) | 0.825 (± 0.096) | 0.874 (± 0.07) | 0.923 (± 0.044) |
|  |  |  | GCN | 0.803 (± 0.022) | 0.786 (± 0.024) | 0.795 (± 0.023) | 0.786 (± 0.023) | 0.844 (± 0.017) | 0.901 (± 0.01) |
|  |  |  | GAT | 0.95 (± 0.054) | 0.945 (± 0.064) | 0.952 (± 0.05) | 0.945 (± 0.055) | 0.96 (± 0.04) | 0.975 (± 0.025) |
|  |  |  | GraphSAGE | 0.96 (± 0.026) | 0.957 (± 0.029) | 0.961 (± 0.022) | 0.955 (± 0.029) | 0.967 (± 0.021) | 0.98 (± 0.013) |
| 50 | Cross-entropy Loss | 32 | MagNet | 0.961 (± 0.014) | 0.957 (± 0.015) | 0.96 (± 0.016) | 0.956 (± 0.015) | 0.968 (± 0.011) | 0.98 (± 0.007) |
|  |  |  | GCN | 0.766 (± 0.012) | 0.748 (± 0.013) | 0.751 (± 0.014) | 0.75 (± 0.013) | 0.816 (± 0.009) | 0.883 (± 0.006) |
|  |  |  | GAT | 0.968 (± 0.007) | 0.965 (± 0.008) | 0.968 (± 0.008) | 0.963 (± 0.007) | 0.973 (± 0.005) | 0.984 (± 0.003) |
|  |  |  | GraphSAGE | 0.958 (± 0.006) | 0.955 (± 0.006) | 0.956 (± 0.006) | 0.954 (± 0.006) | 0.966 (± 0.004) | 0.979 (± 0.003) |
|  |  | 64 | MagNet | 0.95 (± 0.026) | 0.945 (± 0.028) | 0.951 (± 0.024) | 0.943 (± 0.028) | 0.959 (± 0.02) | 0.975 (± 0.012) |
|  |  |  | GCN | 0.763 (± 0.011) | 0.745 (± 0.014) | 0.749 (± 0.014) | 0.746 (± 0.014) | 0.814 (± 0.01) | 0.881 (± 0.006) |
|  |  |  | GAT | 0.965 (± 0.013) | 0.962 (± 0.014) | 0.964 (± 0.015) | 0.96 (± 0.013) | 0.971 (± 0.009) | 0.982 (± 0.006) |
|  |  |  | GraphSAGE | 0.962 (± 0.005) | 0.959 (± 0.006) | 0.961 (± 0.006) | 0.958 (± 0.006) | 0.969 (± 0.004) | 0.981 (± 0.003) |
|  | Focal Loss | 32 | MagNet | 0.924 (± 0.061) | 0.915 (± 0.071) | 0.932 (± 0.046) | 0.913 (± 0.068) | 0.937 (± 0.049) | 0.962 (± 0.03) |
|  |  |  | GCN | 0.759 (± 0.011) | 0.735 (± 0.012) | 0.739 (± 0.013) | 0.74 (± 0.013) | 0.81 (± 0.009) | 0.879 (± 0.006) |
|  |  |  | GAT | 0.96 (± 0.008) | 0.957 (± 0.009) | 0.961 (± 0.009) | 0.955 (± 0.009) | 0.967 (± 0.007) | 0.98 (± 0.004) |
|  |  |  | GraphSAGE | 0.962 (± 0.02) | 0.959 (± 0.021) | 0.962 (± 0.023) | 0.958 (± 0.019) | 0.97 (± 0.015) | 0.981 (± 0.01) |
|  |  | 64 | MagNet | 0.896 (± 0.093) | 0.879 (± 0.114) | 0.911 (± 0.068) | 0.88 (± 0.107) | 0.914 (± 0.077) | 0.948 (± 0.047) |
|  |  |  | GCN | 0.756 (± 0.015) | 0.733 (± 0.016) | 0.739 (± 0.012) | 0.736 (± 0.018) | 0.807 (± 0.013) | 0.878 (± 0.008) |
|  |  |  | GAT | 0.964 (± 0.01) | 0.962 (± 0.011) | 0.965 (± 0.01) | 0.959 (± 0.011) | 0.971 (± 0.008) | 0.982 (± 0.005) |
|  |  |  | GraphSAGE | 0.961 (± 0.017) | 0.957 (± 0.019) | 0.962 (± 0.016) | 0.955 (± 0.02) | 0.968 (± 0.014) | 0.98 (± 0.008) |
| 100 | Cross-entropy Loss | 32 | MagNet | 0.967 (± 0.008) | 0.964 (± 0.009) | 0.967 (± 0.007) | 0.962 (± 0.009) | 0.973 (± 0.006) | 0.983 (± 0.004) |
|  |  |  | GCN | 0.73 (± 0.013) | 0.704 (± 0.016) | 0.709 (± 0.015) | 0.71 (± 0.016) | 0.787 (± 0.011) | 0.864 (± 0.007) |
|  |  |  | GAT | 0.968 (± 0.005) | 0.966 (± 0.005) | 0.969 (± 0.005) | 0.964 (± 0.005) | 0.974 (± 0.004) | 0.984 (± 0.003) |
|  |  |  | GraphSAGE | 0.96 (± 0.008) | 0.956 (± 0.009) | 0.958 (± 0.01) | 0.955 (± 0.009) | 0.968 (± 0.006) | 0.98 (± 0.004) |
|  |  | 64 | MagNet | 0.967 (± 0.007) | 0.964 (± 0.007) | 0.968 (± 0.006) | 0.962 (± 0.008) | 0.972 (± 0.005) | 0.983 (± 0.003) |
|  |  |  | GCN | 0.723 (± 0.009) | 0.693 (± 0.011) | 0.702 (± 0.009) | 0.701 (± 0.015) | 0.78 (± 0.01) | 0.859 (± 0.005) |
|  |  |  | GAT | 0.969 (± 0.005) | 0.967 (± 0.005) | 0.97 (± 0.005) | 0.965 (± 0.005) | 0.974 (± 0.004) | 0.984 (± 0.002) |
|  |  |  | GraphSAGE | 0.955 (± 0.011) | 0.952 (± 0.012) | 0.953 (± 0.013) | 0.951 (± 0.01) | 0.965 (± 0.008) | 0.978 (± 0.005) |
|  | Focal Loss | 32 | MagNet | 0.941 (± 0.028) | 0.935 (± 0.03) | 0.943 (± 0.027) | 0.932 (± 0.031) | 0.952 (± 0.022) | 0.971 (± 0.014) |
|  |  |  | GCN | 0.734 (± 0.012) | 0.706 (± 0.014) | 0.711 (± 0.012) | 0.711 (± 0.014) | 0.788 (± 0.01) | 0.865 (± 0.007) |
|  |  |  | GAT | 0.965 (± 0.005) | 0.962 (± 0.007) | 0.965 (± 0.007) | 0.96 (± 0.006) | 0.971 (± 0.004) | 0.982 (± 0.002) |
|  |  |  | GraphSAGE | 0.961 (± 0.015) | 0.957 (± 0.016) | 0.959 (± 0.016) | 0.956 (± 0.016) | 0.968 (± 0.012) | 0.98 (± 0.007) |
|  |  | 64 | MagNet | 0.954 (± 0.019) | 0.95 (± 0.02) | 0.956 (± 0.017) | 0.947 (± 0.02) | 0.962 (± 0.014) | 0.977 (± 0.009) |
|  |  |  | GCN | 0.727 (± 0.015) | 0.697 (± 0.019) | 0.704 (± 0.014) | 0.705 (± 0.019) | 0.784 (± 0.013) | 0.862 (± 0.008) |
|  |  |  | GAT | 0.963 (± 0.006) | 0.96 (± 0.007) | 0.964 (± 0.006) | 0.958 (± 0.007) | 0.969 (± 0.005) | 0.981 (± 0.003) |
|  |  |  | GraphSAGE | 0.932 (± 0.101) | 0.923 (± 0.12) | 0.939 (± 0.075) | 0.924 (± 0.111) | 0.945 (± 0.081) | 0.966 (± 0.051) |
| 200 | Cross-entropy Loss | 32 | MagNet | 0.972 (± 0.003) | 0.97 (± 0.003) | 0.973 (± 0.003) | 0.968 (± 0.003) | 0.977 (± 0.002) | 0.986 (± 0.002) |
|  |  |  | GCN | 0.699 (± 0.019) | 0.66 (± 0.022) | 0.674 (± 0.016) | 0.675 (± 0.023) | 0.76 (± 0.018) | 0.846 (± 0.012) |
|  |  |  | GAT | 0.961 (± 0.008) | 0.958 (± 0.007) | 0.961 (± 0.007) | 0.956 (± 0.008) | 0.968 (± 0.006) | 0.98 (± 0.004) |
|  |  |  | GraphSAGE | 0.91 (± 0.138) | 0.896 (± 0.171) | 0.915 (± 0.116) | 0.9 (± 0.157) | 0.926 (± 0.118) | 0.953 (± 0.078) |
|  |  | 64 | MagNet | 0.966 (± 0.007) | 0.963 (± 0.008) | 0.966 (± 0.007) | 0.961 (± 0.008) | 0.972 (± 0.006) | 0.983 (± 0.004) |
|  |  |  | GCN | 0.696 (± 0.014) | 0.658 (± 0.015) | 0.668 (± 0.013) | 0.675 (± 0.016) | 0.76 (± 0.012) | 0.845 (± 0.009) |
|  |  |  | GAT | 0.967 (± 0.004) | 0.964 (± 0.004) | 0.968 (± 0.004) | 0.962 (± 0.005) | 0.972 (± 0.003) | 0.983 (± 0.002) |
|  |  |  | GraphSAGE | 0.943 (± 0.043) | 0.938 (± 0.047) | 0.944 (± 0.034) | 0.937 (± 0.049) | 0.954 (± 0.035) | 0.972 (± 0.022) |
|  | Focal Loss | 32 | MagNet | 0.958 (± 0.016) | 0.954 (± 0.018) | 0.96 (± 0.015) | 0.952 (± 0.018) | 0.965 (± 0.013) | 0.979 (± 0.008) |
|  |  |  | GCN | 0.697 (± 0.039) | 0.66 (± 0.059) | 0.681 (± 0.015) | 0.674 (± 0.046) | 0.761 (± 0.032) | 0.848 (± 0.018) |
|  |  |  | GAT | 0.964 (± 0.007) | 0.961 (± 0.008) | 0.965 (± 0.007) | 0.958 (± 0.009) | 0.97 (± 0.006) | 0.981 (± 0.004) |
|  |  |  | GraphSAGE | 0.938 (± 0.072) | 0.934 (± 0.075) | 0.937 (± 0.071) | 0.935 (± 0.068) | 0.952 (± 0.051) | 0.97 (± 0.033) |
|  |  | 64 | MagNet | 0.905 (± 0.143) | 0.892 (± 0.169) | 0.905 (± 0.153) | 0.899 (± 0.141) | 0.926 (± 0.104) | 0.954 (± 0.067) |
|  |  |  | GCN | 0.703 (± 0.013) | 0.673 (± 0.012) | 0.679 (± 0.01) | 0.68 (± 0.015) | 0.765 (± 0.011) | 0.85 (± 0.008) |
|  |  |  | GAT | 0.963 (± 0.007) | 0.96 (± 0.008) | 0.962 (± 0.008) | 0.958 (± 0.007) | 0.969 (± 0.005) | 0.981 (± 0.003) |
|  |  |  | GraphSAGE | 0.964 (± 0.007) | 0.961 (± 0.008) | 0.962 (± 0.008) | 0.96 (± 0.007) | 0.971 (± 0.005) | 0.982 (± 0.003) |

**Note: Results are reported as mean (± standard deviation), #Nbr: number of neighbors in graph construction, #Dim: number of output dimension in graph learning.**

**eTable 7**. Bootstrapped Clustering Results (100 Iterations)

| Representation | K | Jaccard Similarity (±SD) | Silhouette Score (±SD) | Davies-Bouldin Index (±SD) |
| --- | --- | --- | --- | --- |
| MagNet | 3 | 0.72 (±0.12) | 0.29 (±0.09) | 1.03 (±0.19) |
|  | 4 | 0.63 (±0.21) | 0.23 (±0.05) | 1.17 (±0.15) |
| GraphSAGE | 3 | 0.56 (±0.14) | 0.31(±0.09) | 0.99 (±0.24) |
|  | 4 | 0.7 (±0.2) | 0.29 (±0.1) | 0.79 (±0.21) |
|  | 5 | 0.65 (±0.24) | 0.27 (±0.07) | 0.81 (±0.2) |
|  | 6 | 0.65 (±0.24) | 0.23 (±0.05) | 0.96 (±0.22) |
|  | 7 | 0.62 (±0.24) | 0.2 (±0.03) | 1.09 (±0.24) |

**Note: Each iteration trains time series K-means with 90% of the data, tests on 100% of the data, and compares Jaccard overlap with the 100% train/test run. Silhouette Score (SS) and Davies-Bouldin Index (DBI) are reported as averages across 100 iterations.**

**eTable 8.** Pairwise Pearson correlations among the four subphenotypes

|  | Subphenotype 1 & Subphenotype 2 | Subphenotype 1 & Subphenotype 3 | Subphenotype 1 & Subphenotype 4 | Subphenotype 2 & Subphenotype 3 | Subphenotype 2 & Subphenotype 4 | Subphenotype 3 & Subphenotype 4 |
| --- | --- | --- | --- | --- | --- | --- |
| Pearson correlation | 0.929 | 0.823 | 0.890 | 0.608 | 0.978 | 0.510 |

**eTable 9.** Model performance (XGBoost) for predicting subphenotypes based on encounter information

| **Subphenotypes** | **Precision** | **Recall** | **F1-Score** | **Support** |
| --- | --- | --- | --- | --- |
| 1 | 0.589 | 0.807 | 0.681 | 259 |
| 2 | 0.500 | 0.029 | 0.056 | 34 |
| 3 | 0.557 | 0.393 | 0.461 | 112 |
| 4 | 0.551 | 0.380 | 0.450 | 100 |
| macro mean | 0.549 | 0.402 | 0.412 | 505 |

**eFigure 1.** Silhouette score and Davies-Bouldin Index for time series k-means clustering (K = 2 to 10) utilizing embeddings from GAT, GraphSAGE, and MagNet.

**eFigure 2.** Characteristics of AD progression subphenotypes identified by MagNet with time series K-means (K = 3). (a) MCI to AD transition rates for each subphenotype. (b) Kaplan-Meier survival curves stratified by subphenotype. (c) Demographic characteristics of each subphenotype.

**eFigure 3.** Clinical characteristics of AD progression subphenotypes identified by MagNet with time series K-means (K = 3). (a) Heatmap of the top 20 features with the highest prevalence in the cohort across the subphenotypes, along with p-value comparisons between each pair of subphenotypes. (b) Most correlated comorbidities and their prevalence for each subphenotype.

**eFigure 4.** Characteristics of AD progression subphenotypes identified by GraphSAGE with time series K-means (K = 3). (a) MCI to AD transition rates for each subphenotype. (b) Kaplan-Meier survival curves stratified by subphenotype. (c) Demographic characteristics of each subphenotype.

**eFigure 5.** Clinical characteristics of AD progression subphenotypes identified by GraphSAGE with time series K-means (K = 3). (a) Heatmap of the top 20 features with the highest prevalence in the cohort across the subphenotypes, along with p-value comparisons between each pair of subphenotypes. (b) Most correlated comorbidities and their prevalence for each subphenotype.

**eFigure 6.** Characteristics of AD progression subphenotypes identified by GraphSAGE with time series K-means (K = 4). (a) MCI to AD transition rates for each subphenotype. (b) Kaplan-Meier survival curves stratified by subphenotype. (c) Demographic characteristics of each subphenotype.

**eFigure 7.** Clinical characteristics of AD progression subphenotypes identified by GraphSAGE with time series K-means (K = 4). (a) Heatmap of the top 20 features with the highest prevalence in the cohort across the subphenotypes, along with p-value comparisons between each pair of subphenotypes. (b) Most correlated comorbidities and their prevalence for each subphenotype.

**eFigure 8.** Characteristics of AD progression subphenotypes identified by GraphSAGE with time series K-means (K = 5). (a) MCI to AD transition rates for each subphenotype. (b) Kaplan-Meier survival curves stratified by subphenotype. (c) Demographic characteristics of each subphenotype.

**eFigure 9.** Clinical characteristics of AD progression subphenotypes identified by GraphSAGE with time series K-means (K = 5). (a) Heatmap of the top 20 features with the highest prevalence in the cohort across the subphenotypes, along with p-value comparisons between each pair of subphenotypes. (b) Most correlated comorbidities and their prevalence for each subphenotype.

**eFigure 10.** Characteristics of AD progression subphenotypes identified by GraphSAGE with time series K-means (K = 6). (a) MCI to AD transition rates for each subphenotype. (b) Kaplan-Meier survival curves stratified by subphenotype. (c) Demographic characteristics of each subphenotype.

**eFigure 11.** Clinical characteristics of AD progression subphenotypes identified by GraphSAGE with time series K-means (K = 6). (a) Heatmap of the top 20 features with the highest prevalence in the cohort across the subphenotypes, along with p-value comparisons between each pair of subphenotypes. (b) Most correlated comorbidities and their prevalence for each subphenotype.

**eFigure 12.** Characteristics of AD progression subphenotypes identified by GraphSAGE with time series K-means (K = 7). (a) MCI to AD transition rates for each subphenotype. (b) Kaplan-Meier survival curves stratified by subphenotype. (c) Demographic characteristics of each subphenotype.

**eFigure 13.** Clinical characteristics of AD progression subphenotypes identified by GraphSAGE with time series K-means (K = 7). (a) Heatmap of the top 20 features with the highest prevalence in the cohort across the subphenotypes, along with p-value comparisons between each pair of subphenotypes. (b) Most correlated comorbidities and their prevalence for each subphenotype.

**References**

1. *Article Navigation Journal Article Evaluating Single-Cell Cluster Stability Using the Jaccard Similarity Index*.
